# 金属有机框架材料UiO-67富集净化牛奶中孕激素残留

**DOI:** 10.3724/SP.J.1123.2022.04002

**Published:** 2022-08-08

**Authors:** Weiwei SHANG, Decheng SUO, Tong LI, Qiuling DU, Xianhong JIANG, Peilong WANG

**Affiliations:** 中国农业科学院农业质量标准与检测技术研究所, 北京 100081; Institute of Quality Standards and Testing Technology for Agricultural Products, Chinese Academy of Agricultural Science, Beijing 100081, China

**Keywords:** 超高效液相色谱-四极杆/静电场轨道离子阱高分辨质谱, 样品前处理, 固相萃取, 金属有机框架材料, 孕激素, ultra-high performance liquid chromatography-quadrupole/electrostatic field orbitrap high resolution mass spectrometry (UHPLC-Q-Orbitrap HRMS), sample pretreatment, solid phase extraction, metal-organic framework material, progesterone

## Abstract

该文制备了一种比表面积大、稳定性好的金属有机框架材料(MOF)UiO-67,将其用于复杂牛奶基质中痕量孕激素的富集和净化。结合超高效液相色谱-四极杆/静电场轨道离子阱高分辨质谱(UHPLC-Q-Orbitrap HRMS),建立了同时测定牛奶中7种孕激素残留的检测方法。采用X射线光电子能谱(XPS)对UiO-67吸附孕激素后的元素谱图进行测试分析,通过对比结合能和官能团相对含量的变化证明了UiO-67与孕激素间发生了化学作用,并研究了UiO-67对1 mg/L和5 mg/L孕激素的吸附效果。考察并优化了吸附剂用量、洗脱溶剂类型和pH值等关键参数,评价了基质效应对孕激素质谱信号的影响。优化结果显示,选择吸附剂用量为40 mg,在样品溶液pH值为5时,用5 mL丙酮洗脱目标物就可获得满意的回收率。基质效应在20%之内,可忽略不计。在优化条件下,7种孕激素在1~100 μg/L范围内线性相关性良好,相关系数均达到0.99以上,方法的检出限(LOD)和定量限(LOQ)分别为0.06~0.30 μg/L和0.19~1.0 μg/L;在不同添加水平下,回收率为87.10%~105.58%,相对标准偏差为2.66%~9.64%。方法成功应用于实际牛奶样品中孕激素的测定,结果与SN/T 1980-2007标准得到的结果具有良好的吻合度。相比较已报道的牛奶中孕激素前处理技术和检测方法,该方法具有较高的灵敏度和满意的回收率,为今后这类材料在复杂基质中有毒有害物质的富集检测提供了新的借鉴思路。

## Abstract (en)

Progesterone functions as an endocrine-disrupting compound. Imitating endogenous hormones disrupt the animals’ hormone levels. The potential hazard of progesterone in milk cannot be neglected. Thus, research has focused on establishing an efficient and convenient pretreatment and analytical approach. In this study, a metal-organic framework (MOF) material UiO-67 was prepared, which possessed a large specific surface area and excellent stability. It was employed to enrich and purify trace progesterones in a complex milk matrix as a filler to integrate the solid phase extraction column. An approach based on MOF was developed using ultra-high performance liquid chromatography-quadrupole/electrostatic field orbitrap high resolution mass spectrometry (UHPLC-Q-Orbitrap HRMS). This approach could simultaneously determine seven kinds of progesterone residues in milk. The element spectra of UiO-67 were first measured and analyzed using X-ray photoelectron spectroscopy. The chemical interaction between UiO-67 and progesterone was proved by comparing the changes in binding energy and relative contents of functional groups, and the adsorption efficiency of 1 mg/L and 5 mg/L progesterones by UiO-67 was studied. The adsorption efficiencies of UiO-67 for 1 mg/L and 5 mg/L progesterones were 99.73%-99.95% and 88.87%-99.23%, respectively, according to the results. It proved the efficient adsorption of UiO-67 to progesterones and ensured that subsequent studies went smoothly. Furthermore, key parameters, such as the amount of sorbent, elution solvent type, and pH value, were examined and optimized to obtain optimal extraction recovery of the progesterones. Spiked concentrations of 50 μg/L were employed for extraction optimization. All experiments were performed three times. It also evaluated the matrix effect on mass spectrum signal of the progesterones. The optimized results showed that the seven progesterones could be satisfactorily recovered when the amount of adsorbent was 40 mg, pH value of the sample solution was 5, and elution solution was 5-mL acetone. Additionally, the matrix effect of progesterone in the milk sample was <20%. The matrix effect could be neglected using the aforementioned approach to extract and purify progesterones in milk. Finally, the seven progesterones showed good linearity between 1 and 100 μg/L under the optimized conditions, with linear correlation coefficients values >0.99. The limits of detection (LODs) ranged from 0.06 to 0.30 μg/L, and limits of quantification (LOQs) ranged from 0.19 to 1.0 μg/L, respectively. At various concentration levels of progesterones in milk, the recoveries were 87.10%-105.58%, with relative standard deviations of 2.66%-9.64%. Most importantly, the approach was successfully employed to determine progesterone levels in milk samples, with results in good agreement with the standard SN/T 1980-2007. The proposed approach had the advantages of high sensitivity and satisfactory accuracy compared with the reported pretreatment and detection approaches of progesterone in milk. Satisfactory experimental results can be obtained without the calibration by isotope inner standard. Meanwhile, considering the excellent performance of MOF materials in reducing matrix interference in complex samples, such the application of materials offers a new approach. It can be employed to enrich and detect hazards in a complex matrix in the future.

随着国民经济生活水平的提高,人们对蛋白质的需求日益增加,牛奶成为重要的蛋白质补充来源。但近年来的研究发现,牛奶中存在孕激素等多种危害物残留^[1⇓-3]^。孕激素由于其高生物活性和内分泌干扰效应,被认为是内分泌干扰物(EDCs)的典型污染物^[4,5]^。牛奶成分复杂,包括蛋白质、脂肪及碳水化合物等大分子物质,对其中孕激素等痕量危害物的检测一直是一个挑战。因此,针对牛奶中孕激素残留,建立高效、便捷的前处理技术结合准确的检测方法对于保障食品安全具有重要意义。

目前,关于牛奶中孕激素残留检测常用前处理方法包括液液萃取法(liquid-liquid extraction, LLE)、固相萃取法(solid phase extraction, SPE)、加速溶剂萃取法(accelerated solvent extraction, ASE)等。其中,固相萃取法由于其操作简单、分析物预富集因子高、吸附剂可重复使用、使用有机溶剂体积少等优点,在各种萃取技术中得到了广泛的认可^[6]^,相较于液液萃取,SPE具有有机溶剂用量少、提高萃取率、性价比较高等优势^[7]^。固相萃取材料是固相萃取的核心和关键,影响着SPE富集和净化的性能。Guedes-Alonso等^[8]^利用织物相吸附萃取法(fabric phase sorptive extraction, FPSE)代替传统SPE技术提取牛奶中的类固醇激素,结合超高效液相色谱-串联质谱法进行分析,使用内标法定量,方法简便、快速,但萃取率相对较低。

近年来,金属有机框架材料(metal-organic frameworks, MOFs)作为一种新兴功能材料,在气体储存^[9,10]^、传感器^[11]^、吸附分离^[12⇓⇓⇓-16]^、药物传递^[17,18]^和分析预处理^[19⇓-21]^等方面的应用引起了研究人员的广泛兴趣。在不同的分析应用中,特别是在样品预处理中,MOFs由于其结构拓扑不同、孔径可调、永久纳米孔隙度、比表面积高和热稳定性良好,在环境和生物样品的富集、分析等领域得到广泛应用^[22,23]^。Pang等^[24]^制备了金属有机骨架-聚合物整体柱,作为固相萃取柱,成功地实现在线固相萃取并测定中草药中的熊果酸。Jiang等^[25]^采用聚偏二氟乙烯物理包封的方法,将固体颗粒嵌入三聚氰胺泡沫中,制备了基于MIL-101(Cr)的泡沫柱,并作为一种高质量的吸附材料用于植物油中三嗪类化合物的检测。该泡沫柱的制备方法适用于多种金属有机框架材料,并能保持其独特的性能。Rahimpoor等^[26]^首次报道了一种基于3种MOFs(UiO-66、UiO-66-NH_2_和UiO-66-NH_2_@Fe_3_O_4_-SiO_2_)的分析方法,作为填充吸附剂(MEPS)萃取尿液中的反式,反式-1,3-丁二烯-1,4-二羧酸(tt-MA),方法灵敏、快速,可重复使用。目前,传统的固相萃取柱制备工艺非常成熟,在复杂样品中应用广泛。针对孕激素的SPE固相萃取柱主要包括C_18_、Oasis HLB、Prime HLB等,但其填料用量较多,使用时需进行活化、淋洗等步骤,使实验操作繁杂,且无法二次使用。本研究筛选出一种比表面积大、水稳定性好的锆基金属有机框架材料UiO-67,探究了UiO-67与孕激素间发生的化学作用,并将其作为固相萃取柱填料应用于牛奶中孕激素的富集净化,考察了影响孕激素萃取效果的主要参数,结合超高效液相色谱-四极杆/静电场高分辨质谱技术(UHPLC-Q-Orbitrap HRMS)实现了牛奶中7种孕激素残留的高灵敏测定。

## 1 实验部分

### 1.1 仪器与试剂

Vanquish Flex UHPLC Systems超高效液相色谱、Q-Exactive四极杆-静电场轨道离子阱高分辨质谱仪、热电喷雾电离(heatable electrospray source ion, HESI)源、K-Alpha X射线光电子能谱(XPS)(美国Thermo公司), N-EVAP-112氮吹仪(美国Organomation公司), 3K15高速冷冻离心机(美国Sigma公司), Smart Lab3 X射线衍射仪(XRD,日本理学公司), Tensor Ⅱ傅里叶变换红外光谱仪(FT-IR,德国Bruker公司), SU 3500扫描电子显微镜(SEM,日本日立公司), Milli-Q超纯水仪(法国Merck公司)。

7种孕激素标准品(化学结构式见图1):美仑孕酮(melengestrol, MLG)、甲地孕酮(megestrol, MG)、17*α*-羟孕酮(17*α*-hydroxyprogesterone, 17*α*-HPT)、左炔诺孕酮(levonorgestrel, LG)、孕酮(progesterone, PT)均购自德国Dr. Ehrenstorfer公司,烯丙孕素(altrenogest, AT)购自美国TargetMol公司,甲羟孕酮(medroxyprogesterone, MP)购自上海源叶生物科技有限公司,以上均为纯品型粉末,纯度大于98%。氯化锆(ZrCl_4_)购自上海阿拉丁生化科技股份有限公司,4,4'-联苯二甲酸购自北京华威锐科化工有限公司,无水硫酸镁购自北京易丰源生物科技有限公司,所用甲酸、乙酸、甲醇、乙腈、丙酮、*N*,*N*-二甲基甲酰胺(DMF)(HPLC级,德国CNW公司),实验用水为Milli-Q纯化后的超纯水。

**图1:**
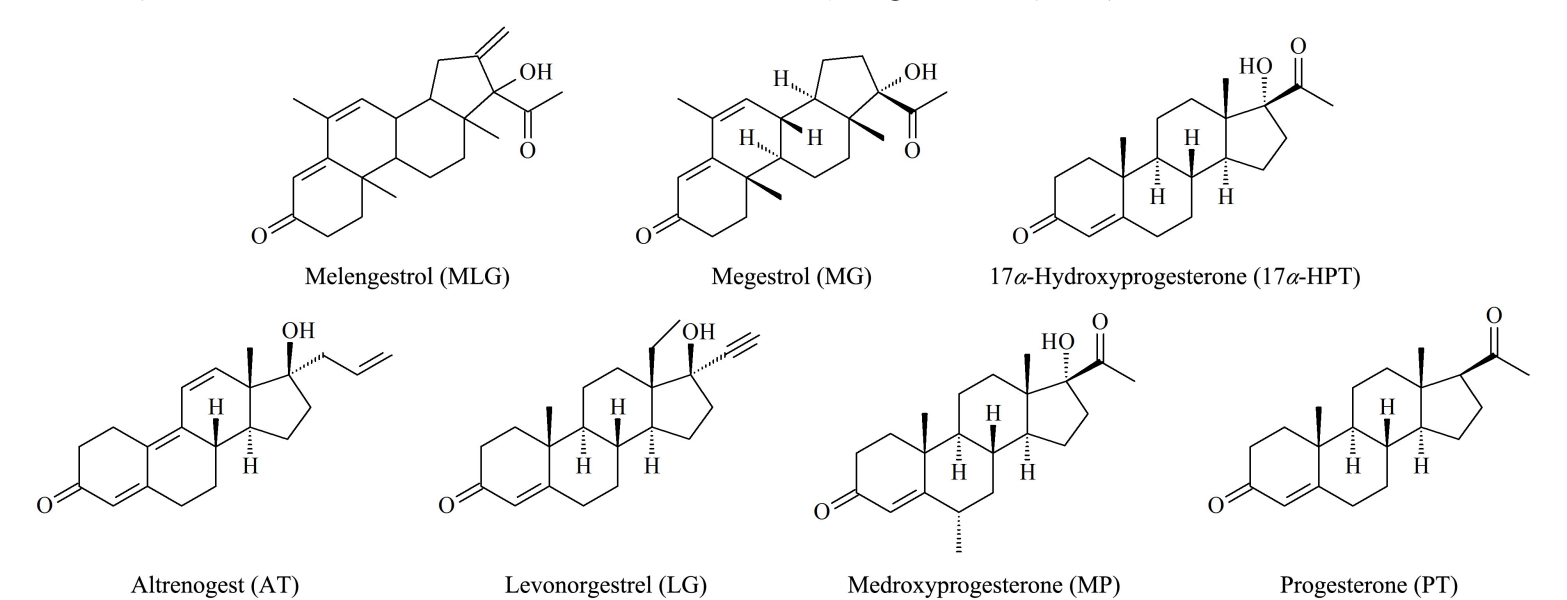
分析物的化学结构式

储备液的配制:分别称取20 mg标准品粉末,加入40 mL甲醇溶解,各配制成质量浓度为500 mg/L的单标准储备液。各取适量500 mg/L单标准储备液用甲醇稀释为10 mg/L的孕激素混合标准工作液,备用。以上所有标准液均于-20 ℃冷藏保存。

### 1.2 材料的合成

在文献^[27]^基础上,采用溶剂热法合成UiO-67。准确称量ZrCl_4_ 46.6 mg与4,4'-联苯二甲酸48.4 mg于聚四氟乙烯内衬反应釜中,向其中加入20 mL *N*,*N*-二甲基甲酰胺后,放入超声清洗器中超声混匀,然后加入0.2 mL乙酸使其充分混合后,放入120 ℃烘箱中反应24 h。用DMF和丙酮各清洗3次后,在50 ℃烘箱中过夜干燥。

### 1.3 固相萃取柱的制备

加入垂直放置的、装有多孔聚乙烯筛板的5 mL聚丙烯管中,一次性装入自制材料UiO-67 40 mg,多次轻敲管壁使其分布均匀,将另一片多孔聚乙烯筛板放置在填料上方后压紧,使填料上方表面平齐并密实,制成40 mg/5 mL固相萃取小柱。

### 1.4 UiO-67对孕激素的吸附性研究

向3 mL含有1 mg/L和5 mg/L的7种孕激素溶液中分别加入3 mg UiO-67,在28 ℃, 240 r/min设定条件下摇床振荡30 min,使孕激素被UiO-67充分吸附,然后离心1 min,取上清液1 mL过滤膜(尼龙,0.22 μm),使用UHPLC-Q-Orbitrap HRMS进行检测。吸附效率=(1-孕激素吸附后测得峰面积/孕激素吸附前测得峰面积)×100%。

### 1.5 样品前处理

#### 1.5.1 样品的提取

用移液枪准确吸取5 mL牛奶于50 mL离心管中,向其中加入10 mL乙腈,室温下超声提取15 min,于4 ℃下离心10 min,将提取液全部倒入盛有2 mg无水硫酸镁的离心管中,涡旋后离心10 min,取上层清液于试管中,在50 ℃下氮吹至近干后,用5 mL pH=5的磷酸盐缓冲液复溶,待净化。

#### 1.5.2 样品的净化

取全部复溶液过UiO-67填装的固相萃取小柱,以1 mL/min的流速缓慢通过固相萃取柱,使其充分吸附,然后加入5 mL丙酮通过固相萃取小柱进行洗脱,收集全部洗脱液,在50 ℃下氮吹至近干后用0.5 mL乙腈-水(1∶1, v/v)复溶,涡旋混匀后过滤膜,进样体积为5 μL,使用UHPLC-Q-Orbitrap HRMS检测。

### 1.6 仪器条件

#### 1.6.1 液相色谱条件

色谱柱:ACQUITY UPLC BEH C_18_(100 mm×2.1 mm, 1.7 μm);流动相:A为0.1%甲酸水溶液,B为乙腈;柱温:40 ℃;流速:0.4 mL/min;进样量:5 μL。梯度洗脱条件:0~0.5 min, 30%B; 0.5~1.0 min, 30%B~45%B; 1.0~8.0 min, 45%B~90%B; 8.0~8.8 min, 90%B; 8.8~10.0 min, 90%B~30%B。

#### 1.6.2 质谱条件

采用HESI源,7种孕激素均采用正离子扫描模式,平行反应监测(PRM)模式;鞘气流速:40 Arb;辅助气流速:10 Arb;喷雾电压:3.5 kV;毛细管温度:320 ℃;透镜电压:55 V;辅助气加热温度:300 ℃;容纳离子数目:2.0×10^4^;分辨率:30000;隔离窗口:*m/z* 2.0;离子注入时间:50 ms;脱孔剂气和锥孔气均为氮气。除了甲地孕酮的碰撞能量为35 eV,其余均设置为30 eV。目标化合物的其余质谱参数见表1, 7种孕激素的色谱图见图2。

**表1:** 目标化合物的保留时间和质谱参数

Compound	Retention time/min	Precursor ion (m/z)	Product ions (m/z)
MLG	4.61	355.23	279.18^*^/337.22
MG	4.68	343.23	267.18^*^/325.22
17α-HPT	4.99	331.21	97.07^*^/109.07
AT	5.07	311.21	227.15^*^/269.16
LG	5.23	313.22	109.20^*^/245.30
MP	5.67	345.24	123.08^*^/97.07
PT	5.75	315.24	97.07^*^/109.07

* Quantitative ion.

**图2:**
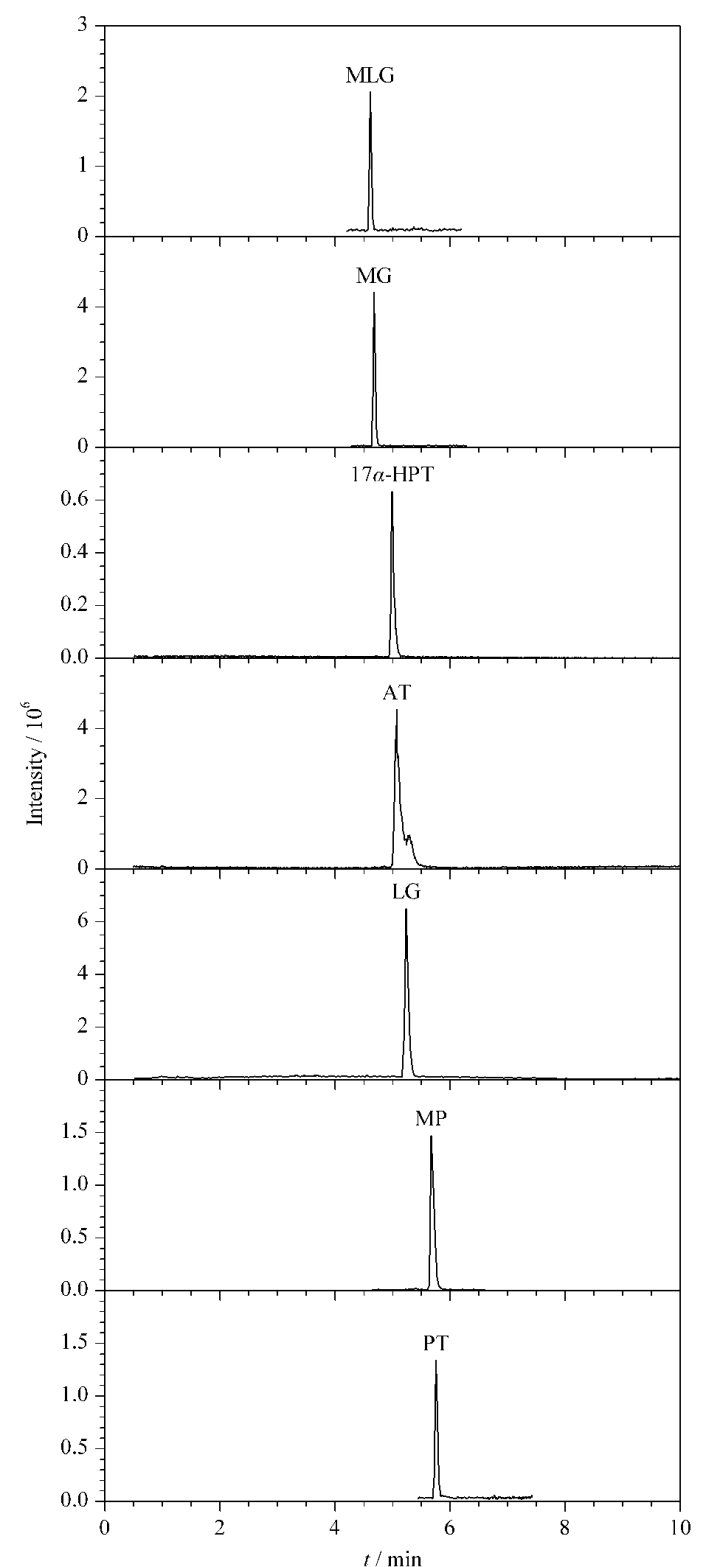
7种孕激素的选择离子流色谱图

## 2 结果与讨论

### 2.1 材料表征

采用扫描电子显微镜、X射线衍射光谱仪、傅里叶变换红外光谱仪对获得的UiO-67进行表征。

如图3a所示,UiO-67的形貌为八面体,尺寸约为84 nm,边长约为60 nm。在5°~40°范围内模拟了UiO-67的粉末X射线衍射图(见图3b),在2*θ*≈5°、6°、12°时的衍射峰和先前文献^[28]^报道吻合;红外光谱图(见图3c)表明,在波段为660 cm^-1^和770 cm^-1^处出现Zr-O键,1385 cm^-1^附近出现Zr-OH键,1500~1650 cm^-1^处出现C-O-Zr键,这与模拟的UiO-67红外光谱图一致,说明材料已被成功制备。

**图3:**
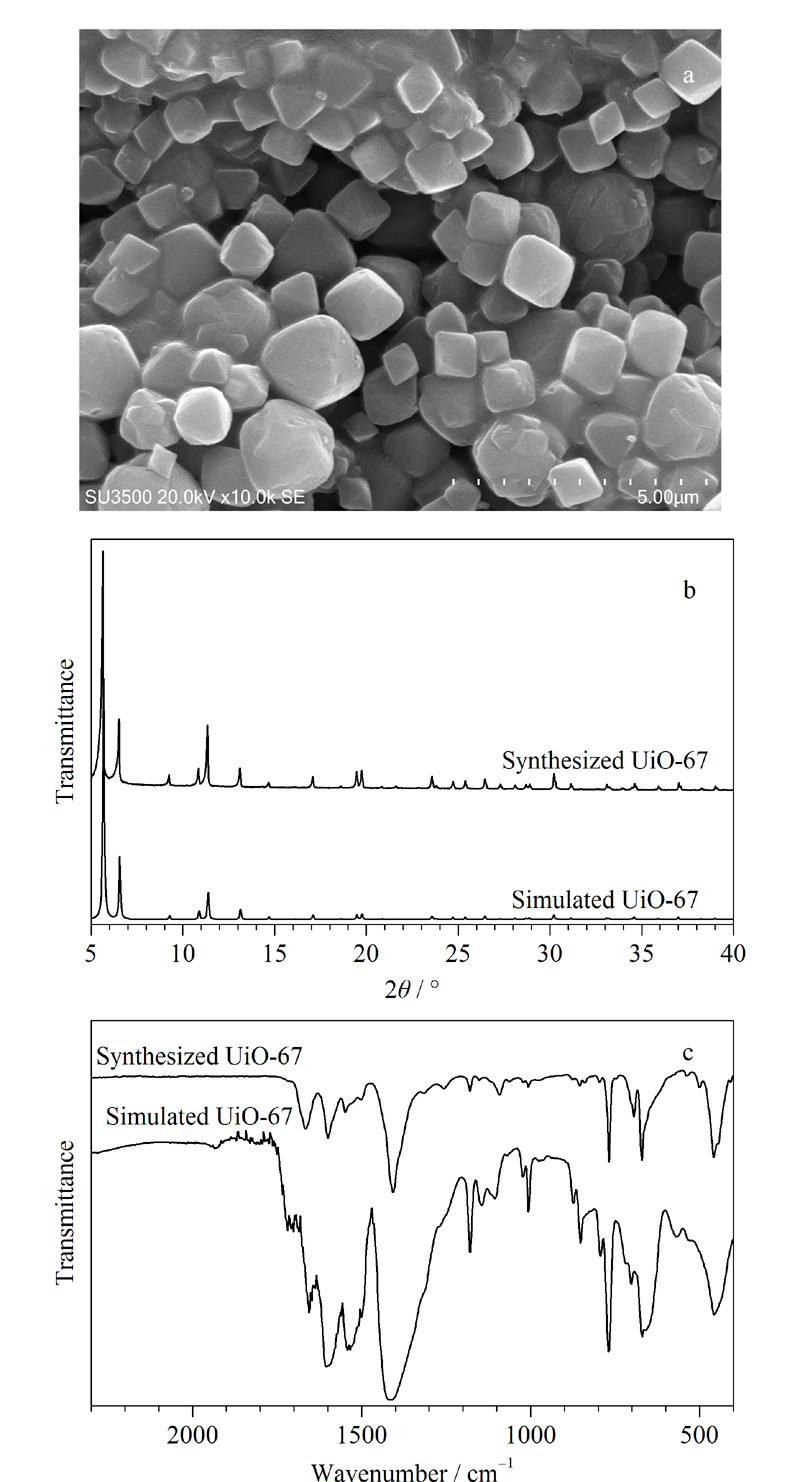
UiO-67的(a)扫描电镜图、(b)X射线衍射光谱图和(c)红外光谱图

此外,采用X射线光电子能谱仪对UiO-67吸附孕激素前和吸附后进行测试,吸附前后Zr、C、O的谱图发生变化。与吸附前相比,UiO-67吸附孕激素后,Zr 3*d*的谱峰向右稍微偏移,Zr 3*d*_5/2_的结合能由182.62 eV移至182.29 eV, Zr 3*d*_3/2_的结合能由185.0 eV移至184.67 eV,说明孕激素与Zr团簇间存在相互作用,Zr作为得电子方发生了化学吸附;UiO-67中C 1*s*谱可分为3个峰,分别对应官能团C-C/C=C、C-O和C=O,孕激素本身结构含有C-C、C=C,所以当UiO-67吸附孕激素后,C-C/C=C相对含量明显增加,由74.20%增加至87.67%,而C-O和C=O的相对含量减少了13.46%;对比UiO-67吸附孕激素前后的O 1*s*谱图变化,各个峰略有偏移,整体结合能减少,Zr-O-Zr、Zr-OH、O-C=O的相对含量也均有减少,而Zr-OH_2_的相对含量增加了17.29%,说明UiO-67与孕激素之间存在化学作用。

### 2.2 UiO-67对孕激素的吸附性

向等体积、不同含量的(1 mg/L和5 mg/L)孕激素混合标准溶液中分别加入等量的UiO-67进行吸附实验,结果如图4所示,UiO-67对1 mg/L的孕激素溶液吸附效率为99.73%~99.95%,对5 mg/L的孕激素溶液吸附效率相较之略低,为88.87%~99.23%,充分证明了这种材料对孕激素的高效吸附性,从而保证在较低孕激素浓度水平下能够顺利进行实验。

**图4:**
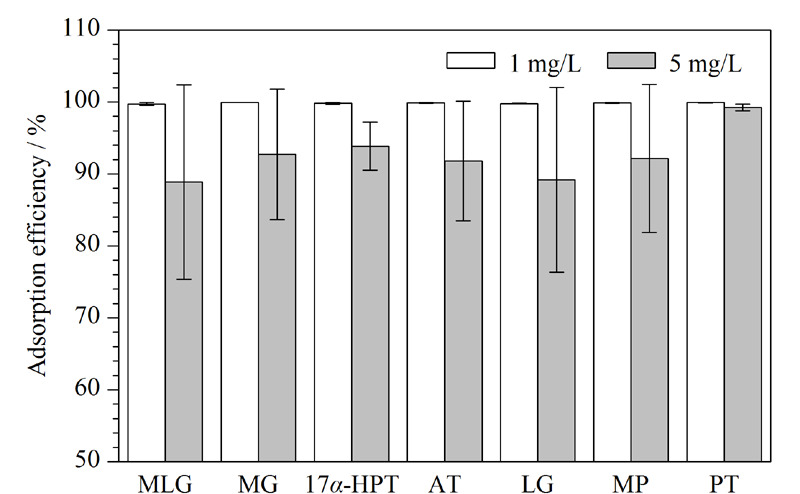
UiO-67对孕激素的吸附效率(*n*=3)

### 2.3 固相萃取条件优化

#### 2.3.1 吸附剂用量

选取不同质量的吸附剂(30、40、50、60 mg)分别填装固相萃取柱,研究吸附剂用量对孕激素回收率的影响。结果如图5a所示,吸附剂用量为30 mg时,孕激素回收率为45.73%~88.74%,回收率较低,可能由于磷酸盐离子堵塞孔道导致吸附不完全;当吸附剂用量增加至40 mg时,回收率可达到90%以上;继续增加用量至50 mg和60 mg,孕激素回收率明显下降,可能因为洗脱溶剂用量不足,难以充分洗脱。由此可见,吸附剂用量为40 mg时即可以满足孕激素的充分萃取,用量过少或过多会导致萃取率较低。

**图5:**
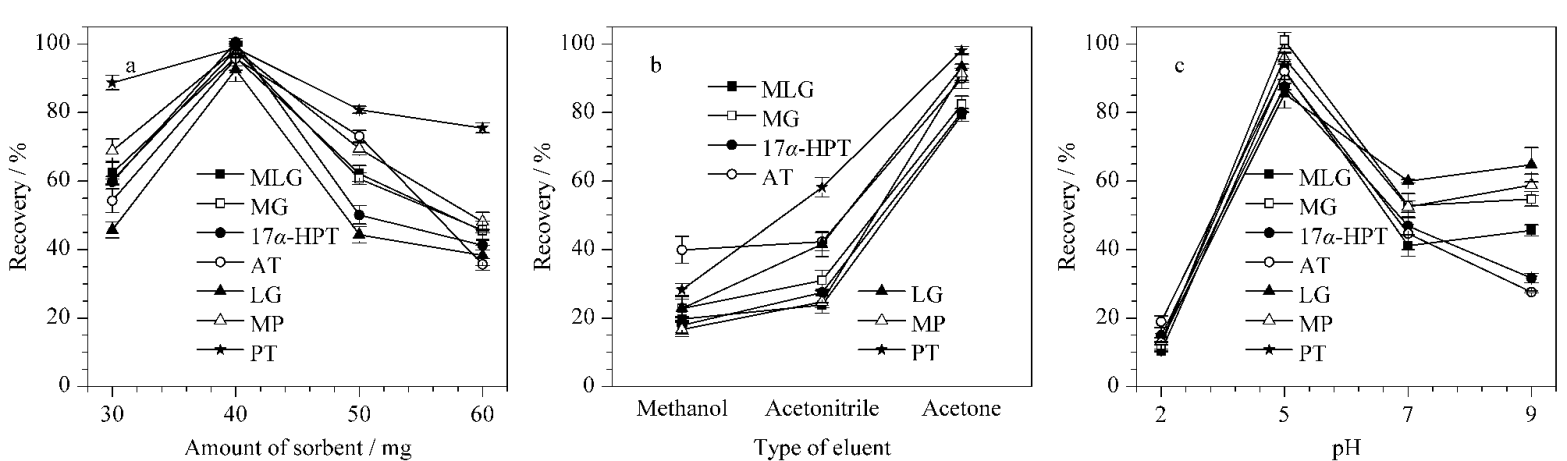
(a)吸附剂用量、(b)洗脱溶剂类型和(c)pH值对孕激素萃取回收率的影响(*n*=3)

#### 2.3.2 洗脱溶剂类型

这7种孕激素属于极性化合物,结合它们的化学结构来看,含有羰基、羟基、甲基,同时具备了亲水和疏水基团,但激素化合物不溶于水,故本实验选择了强极性溶剂进行洗脱,如甲醇、乙腈、丙酮(见图5b)。显而易见,用丙酮洗脱后的孕激素回收率最好,这可能由于丙酮渗入材料内部使其孔径膨胀^[29]^,使目标物顺利从材料中洗脱下来,因此洗脱溶剂选择丙酮。

#### 2.3.3 溶液pH值

牛奶本身呈弱酸性,正常pH值在6~7之间,而样品溶液的pH值对于目标物的萃取起到非常重要的作用,因此考察了pH在2~9范围内UiO-67对孕激素的萃取效果(见图5c)。样品溶液pH=2时,孕激素的萃取率低至20%左右;当溶液pH上调至5时,回收率显著增加至85%以上;继续调节溶液pH为7和9时,回收率显著降低。可能因为溶液pH过高或过低,会使UiO-67和孕激素同时带负电荷或正电荷,产生静电排斥作用。因此,选取pH=5作为最佳实验参数进行后续实验。

### 2.4 基质效应

在UHPLC-Q-Orbitrap HRMS检测中,基质效应会造成背景干扰信号强,掩盖痕量有机化合物的出峰,容易出现假阳性目标化合物,从而影响检测结果的准确性。若想在检测方法中消除基质效应,必须对基质效应的强弱进行评估。本实验通过比较标准曲线斜率大小,来评估基质效应强弱。

将标准工作液用甲醇依次稀释成不同质量浓度(1、2、20、50、100 μg/L)的标准溶液,然后按照上述方法,用空白牛奶提取液将标准工作液稀释成不同质量浓度的标准溶液,按照以上建立的分析方法进行检测。以7种孕激素的标准溶液浓度为横坐标,孕激素的色谱峰面积为纵坐标,分别在1~100 μg/L范围内绘制标准曲线,根据公式ME=(*A*-*B*)/*A*×100%计算基质效应。其中,*A*代表纯溶剂标准曲线斜率;*B*代表基质匹配标准曲线斜率。若ME>0,表现为基质抑制效应,若ME<0,表现为基质增强效应。当0≤|ME|≤20%时,说明基质对信号干扰较低,可忽略不计;当20%<|ME|<50%时,表现为中等强度的基质干扰,而当|ME|≥50%,则为强基质干扰。牛奶中美仑孕酮、左炔诺孕酮、孕酮表现为基质抑制效应,甲地孕酮、17*α*-羟孕酮、烯丙孕素、甲羟孕酮表现为基质增强效应,但|ME|均在0%~20%内,基质对孕激素信号干扰较低(见图6)。牛奶中的孕激素经过提取与净化,基质效应可忽略不计,无需使用基质匹配标准曲线定量。

**图6:**
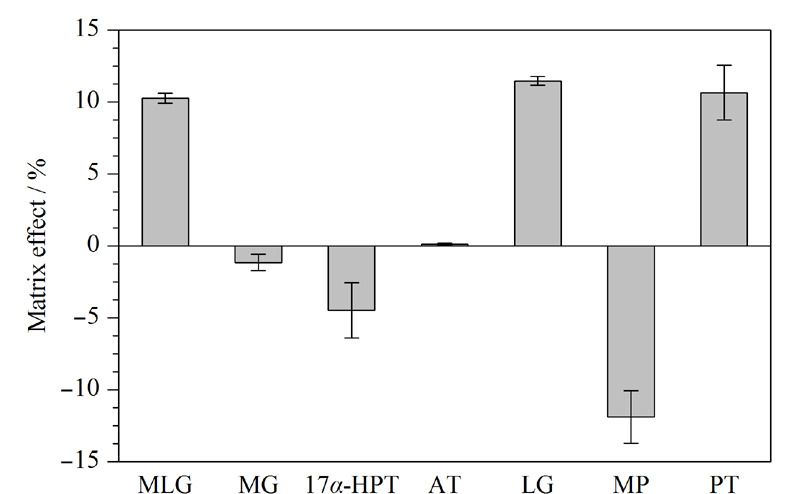
孕激素在牛奶中的基质效应(*n*=3)

### 2.5 方法学验证

#### 2.5.1 线性范围、检出限和定量限

在1~100 μg/L孕激素混合溶液范围内,7种孕激素的线性方程、相关系数、检出限和定量限如表2所示。7种孕激素线性相关系数(*R*^2^)均大于0.99,说明孕激素在此范围内线性相关性良好。在空白基质溶液中添加不同浓度的激素化合物,从低浓度到高浓度依次进行上机检测,以信噪比(*S/N*)≥3确定方法的检出限为0.06~0.30 μg/L,以*S/N*≥10确定方法的定量限为0.19~1.0 μg/L。

**表2:** 孕激素的线性方程、相关系数、检出限和定量限

Compound	Linear equation	R^2^	LOD/ (μg/L)	LOQ/ (μg/L)
MLG	y=4.46×10^5^ x+1.08×10^5^	0.9992	0.30	1.0
MG	y=8.29×10^5^ x+2.33×10^5^	0.9981	0.06	0.19
17α-HPT	y=1.94×10^5^ x-2.52×10^5^	0.9993	0.25	0.84
AT	y=1.06×10^5^ x+1.97×10^5^	0.9972	0.30	1.0
LG	y=1.25×10^4^ x+4.15×10^5^	0.9964	0.17	0.58
MP	y=4.53×10^4^ x-4.95×10^4^	0.9964	0.27	0.90
PT	y=7.32×10^4^ x+7.52×10^5^	0.9998	0.25	0.84

*y*: peak area; *x*: mass concentration, μg/L.

#### 2.5.2 回收率、精密度

在牛奶中分别添加1 μg/L和5 μg/L水平的7种孕激素混合标准溶液,按照上述优化好的样品前处理方法进行添加回收试验,然后上机检测。检测结果见表3,在1 μg/L和5 μg/L孕激素添加水平下,回收率为87.10%~105.58%,相对标准偏差为2.66%~9.64%。

**表3:** 孕激素在不同添加水平下的回收率和精密度(*n*=3)

Compound	1 μg/L			5 μg/L		
	Recovery/%	RSD/%		Recovery/%		RSD/%
MLG	99.64	2.71		105.58	5.86	
MG	87.10	3.68		99.79	6.52	
17α-HPT	101.78	5.54		101.45	9.64	
AT	92.63	8.27		101.50	8.23	
LG	93.55	7.46		93.83	7.98	
MP	96.59	2.66		94.10	7.05	
PT	97.36	6.63		98.40	7.08	

### 2.6 实际样品检测

在超市购买国内牛奶(1号、2号)和国外牛奶(3号、4号),同时按照上述优化的固相萃取条件进行处理,选取良好的仪器状态,使用UHPLC-Q-Orbitrap HRMS测定,结果见表4。该方法检测到牛奶中含有内源性激素(17*α*-羟孕酮、孕酮)和人工合成类激素(甲地孕酮、左炔诺孕酮、甲羟孕酮),未检测到美仑孕酮和烯丙孕素,可能喂养奶牛的饲料或饮用水中使用了激素类药物,导致牛奶中激素残留。孕酮在不同品牌的牛奶中检测浓度较高,这与奶牛处于的生长阶段相关。孕酮的检测结果在14.32~20.12 μg/L范围内,与标准SN/T 1980-2007得到的检测结果比较吻合,更具有说服力。而且相较于标准方法,该方法使用外标法定量,简化操作步骤,是一项有参考意义的前处理和检测方法。此外,饲养动物过程中,值得注意的是孕酮含量以及人工合成类激素的使用。

**表4:** 本方法与标准方法对牛奶中孕激素含量的测定

Compound	This work					SN/T 1980-2007			
	Sample 1	Sample 2	Sample 3	Sample 4		Sample 1	Sample 2	Sample 3	Sample 4
MLG	N/A	N/A	N/A	N/A		N/A	N/A	N/A	N/A
MG	1.46	1.05	1.08	0.98		1.13	0.98	0.89	0.92
17α-HPT	0.85	0.50	0.43	0.49		0.88	0.73	0.35	0.62
AT	N/A	N/A	N/A	N/A		N/A	N/A	N/A	N/A
LG	9.29	15.72	9.50	13.23		10.11	16.58	11.35	14.46
MP	0.78	0.97	0.89	0.73		0.74	0.86	0.64	0.63
PT	14.32	18.57	15.20	20.12		15.82	20.02	17.24	18.45

N/A: no data.

### 2.7 与其他方法比较

将之前报道的牛奶中孕激素前处理方法^[30⇓⇓⇓⇓⇓⇓-37]^与该方法进行比较,对方法的检出限、定量限和回收率以及优缺点进行汇总,如表5所示,该方法检出限较低,且能够获得满意的回收率,具有高效、准确性好、灵敏度高等优点,可以实现牛奶中微量孕激素的富集检测。

**表5:** 本方法与其他方法的比较

Adsorbent	Detection method	Number of analyte	LOD/ (μg/L)	Recovery/ %	Advantage	Disadvantage	Ref.	
Prime HLB	UHPLC-Q-Orbitrap HRMS	21	0.050- 0.30	80.70- 108.30	good recovery	high solvent consumption	[30]	
Oasis HLB	UHPLC-MS/MS	1	0.027	97.29- 102.71	low LOD and good recovery	complicated process and few number of analyte detected	[31]	
HLB	UHPLC-MS/MS	7	0.10- 0.30	70.50- 97.50	good recovery	high LOD	[32]	
C_18_, Oasis HLB	UHPLC-MSD	3	0.50- 1.0	73.40- 86.70	low matrix effect	high LOD and low recovery	[33]	
PSA and acid aluminum oxide	UHPLC-QTOF-MS	4	0.070- 0.30	77.10- 99.80	low LOD and good recovery	troublesome experiment procedure	[34]	
Oasis HLB	LC-MS/MS	3	0.15- 0.20	86.00- 91.20	good recovery	complicated process	[35]	
Fe/CNT-SrTiO_3_	HPLC	1	0.033	64.24- 113.49	low LOD and short time consuming	few number of analyte detected	[36]	
HLB	UHPLC-MS/MS	1	0.50	82.20- 103.00	good recovery	complicated process and few number of analyte detected	[37]	
UiO-67	UHPLC-Q-Orbitrap HRMS	7	0.060- 0.30	87.10- 105.58	good recovery, low LOD, little material consumption	long material preparation time	this work	

MSD: mass spectrometric detector; QTOF: quadrupole-time of flight.

## 3 结论

本研究利用UiO-67作为固相萃取填料,结合UHPLC-Q-Orbitrap HRMS成功建立了同时富集测定牛奶中7种孕激素的分析方法。其中UiO-67作为固相萃取柱材料稳定性好,生产成本较低,应用该方法可减少固相萃取柱的活化步骤,既能节省时间,又防止溶剂浪费,提高实验效率。而且通过与传统前处理方法比较,该方法性能优异,能够实现牛奶中孕激素高灵敏、多残留检测,为牛奶中其他潜在危害物的富集检测提供新的参考。
